# Dual-Task Interference in Children with Down Syndrome and Chronological and Mental Age-Matched Healthy Controls

**DOI:** 10.3390/children9020191

**Published:** 2022-02-02

**Authors:** Benjamin Holfelder, Thomas Jürgen Klotzbier, Nadja Schott

**Affiliations:** Department of Sport and Exercise Science, Institute of Sport Psychology and Movement Performance, University of Stuttgart, 70174 Stuttgart, Germany; thomas.klotzbier@inspo.uni-stuttgart.de (T.J.K.); nadja.schott@inspo.uni-stuttgart.de (N.S.)

**Keywords:** intellectual disability, executive function, motor-cognitive interference, dual-task walking, verbal fluency

## Abstract

Background. On the assumption that motor actions result from the interaction between cognitive, perceptual, and neurological mechanisms, neuromotor dysfunction–such as in children with Down Syndrome (DS)–is expected to affect the central coordination processes required for dual-task (DT) performance. There are few dual-task (DT) studies in individuals with DS, so the current study examined the effects of dual-tasking (DT) on walking performance in children with DS. Method. In this study, a motor-cognitive DT was used in 12 children with DS (10.5 ± 1.08 years, 6 female), 12 typically developed (TD) children with the same mental age (TD-MA: 5.98 ± 1.21 years, 6 female), and 12 with the same chronological age (TD-CA: 10.5 ± 1.07 years, 6 female). Children were asked to enumerate animals for one minute while walking straight ahead. Results. All groups showed lower performances under the DT condition than the single-task (ST) condition. Children with DS appear to have the most difficulties in motor and cognitive tasks and ST- and DT-conditions. Concerning the DT costs (DTC), difficulties were mainly observed with the motor task, with motor DTC being greater than cognitive DTC. Conclusion. The interplay of different systems seems to play a crucial role in walking, especially in children with DS. DT walking paradigms with directional changes are recommended for future studies, as this is more appropriate for the everyday demands of children.

## 1. Introduction

Dual tasks (DT) are proposed as a valid approach to investigate the interaction between cognitive and motor domains in individuals with neuromotor disorders such as Down syndrome (DS) [1,2] or Developmental Coordination Disorder (DCD) [3]. The cross-domain effects of impairments in executive functions (EF) play a decisive role in deficits in motor performance, particularly in locomotion. EFs are often divided into cognitive flexibility, inhibition, and working memory [4]. Although most studies in childhood and adolescence show a more or less linear development of EF, developmental trajectories in typically developed (TD) children are partially inconsistent [5], with large inter-individual variability in age-related rates of change. Looking at performance in motor-cognitive interference tasks, Al-Yahya and colleagues [6] showed that especially mental tracking tasks (internal confounders such as counting backward or verbal fluency tasks) cause significant DT costs (DTC). For example, Krampe et al. [7] examined TD children between 9 and 11 years of age using a similar procedure to the one used in the present study. The children were asked to complete a word fluency task while walking. The authors reported greater difficulties in the walking task and fewer category examples generated in a semantic fluency task under DT conditions. Boonyong and colleagues [8] compared young (5–6 years) and older (7–16 years) children and adolescents, respectively. The authors showed a developmental trend in attentional resources needed to control locomotion. Their results suggest that locomotion is not characterized by automated processes in children between 5 and 10 years old, and age-related effects in performance under DT conditions are more evident in younger TD children.

Considering the motor and cognitive domains separately, individuals with DS show difficulties in both domains compared to TD [9,10]. A recent meta-analysis by Tungate and Conners [10] revealed heterogeneous results when comparing the EF of DS and TD in age-matched individuals. Here, individuals with DS show EF difficulties, particularly in verbal working memory/short-term memory subdomains. However, the effect sizes are smaller for visual working memory/short-term memory and inhibition. Studies on motor-cognitive interference in children with neuromotor disorders and/or disabilities have only recently come into focus [11,12,13], especially studies in children with intellectual disabilities such as DS are rare [2]. To date, there is only one study conducted in children with DS that has investigated motor-cognitive interference with an ecological valid locomotion task [1]. Regarding proportional DTC, the study shows that children with DS have greater difficulties in the motor domain under DT conditions. A possible reason for the limited number of studies could be the low mental age of children with DS, so DT paradigms seem too demanding. Thus, the extent of interaction between cognitive profiles and motor control in children with DS is still largely unexplored [2,12].

The present study examines the effects of a secondary verbal fluency task on walking performance in TD children (matched for mental age (MA) and chronological age (CA)) and children with DS. Based on the assumption that motor actions result from the interaction between cognitive, perceptual, mechanical, and neurological mechanisms, neuromotor dysfunction is thought to impair the central motor coordination processes required to perform DTs, increasing the DTCs and limiting DT performance. Furthermore, due to children’s still developing attention resources, we predicted that younger children have more pronounced problems under DT conditions [7,8,9,10]. This is especially true for children with DS [1].

## 2. Methods

### 2.1. Participants

Twelve children (*n* = 6 female; 10.5 ± 1.08 years) with DS, 12 chronologically-age-matched (TD-CA; *n* = 12, 6 female; 10.5 ± 1.07 years) and 12 mental-age-matched controls (using the PPVT-IV [14]; TD-MA; 6 female; 5.98 ± 1.21 years) participated in this study (Table 1). Children with neuro-musculoskeletal disorders and/or comorbidities (e.g. cerebral palsy, autism spectrum disorders, blindness, deafness) were excluded from the present study. All TD children were free of physiological impairments and developmental delays. These inclusion criteria were verified by asking the parents and the educators. All assessments were conducted in accordance to the Declaration of Helsinki. The study was reviewed and approved by the University of Stuttgart (AZ 21-020). The children were asked for their willingness and consent to participate in the study. The participant’s legal guardian respectively the next of kin provided written informed consent to take part in this study. Neither the participants nor the legal guardians of the children received any incentive for participating in the study.

#### Matching Procedure

A TD child was assigned to the TD-CA group if the CA was within the 4-month range of the children with DS. A TD child was assigned to the TD-MA control group if the raw score in the PPVT-IV was less than 4 standard deviation points away from the corresponding mean score of the children with DS. 

### 2.2. Single- and Dual-Tasks

In the cognitive single-task condition (ST), the children had to name as many animals as possible within one minute while sitting. In the motor ST condition, the children had to walk back and forth a 10-m distance at a comfortable, self-selected walking speed for one minute. In the DT condition, the children had to perform both tasks simultaneously for one minute. The number of words and the distance covered in meters while walking was recorded.

### 2.3. Experimental Procedure

Parents/next of kin signed the informed consent form upon arrival at the Cognitive and Motor Research Laboratory. First, the children completed the PPVT-IV to divide the groups into CA and MA. Then, the STs and DTs were explained verbally before a practical demonstration was provided. The test duration was about 25 min. In order to ensure optimal cognitive performance and to avoid overload, sufficient breaks were allowed for all children between the different conditions. The testing room was quiet and bright, allowing a 10-m distance to be walked. There was a table with chairs in the rooms so that the ST could be performed in a sitting position to achieve the greatest possible standardization. The ST conditions were randomized. The DT was performed following the ST. The number of correctly recited animals and the distance walked in ST and DT was recorded.

### 2.4. Data Analysis

All statistical analyses were performed using SPSS v.25 (SPSS, Chicago, IL, USA). Motor and cognitive DTCs were calculated as follows [15]:

DTC = (DT performance – ST performance))/ST performance) × 100

2 × 2 ANOVAs with repeated measures were calculated with the group as a fixed factor and ST and DT as dependent variables for motor and cognitive performance to analyze the differences between the groups in motor and cognitive performance under ST and DT conditions. In case of significant results, post hoc tests (Bonferroni correction) were calculated to determine which factor levels differed significantly from one another [16]. An alpha value of 0.05 was used for all statistical tests [17]. Effect sizes for all ANOVAs were reported using partial eta-squared (*ɳ*^2^_p_). There were no missing data.

## 3. Results

**Figure 1:**
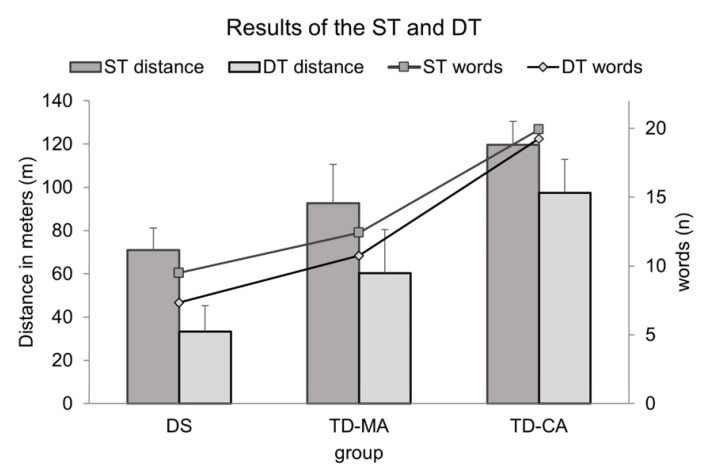
Results for the walking distance and the number of the recited animals for ST and DT conditions in DS, TD-MA, and TD-CA children. Note: DT = dual-task; ST = single-task; DS = Down Syndrome; TD-CA = typically developing children of the same chronological age; TD-MA = Typically developing children of the same mental age.

The 2 × 2 ANOVA with repeated measures showed a significant effect for condition, *F*(1,33) = 113, *p* < 0.001, *ɳ*^2^_p_ = 0.774, with longer distances traveled for the ST condition compared to the DT condition. The group by condition interaction tended to be significant, *F*(2,33) = 2,49, *p* = 0.098, *ɳ*^2^_p_ = 0.131, indicating the best performance in both conditions for the TD-CA group compared to the TD-MA and the children with DS. All groups differed significantly, with children with DS covering the shortest distances and TD-CA children covering the longest distance (Figure 1).

Significant group differences can also be found with respect to the number of recited words under both the ST condition, *F*(2,33) = 12.8, *p* < 0.001, *ɳ*^2^_p_ = 0.430, and the DT condition, *F*(2,33) = 22.5, *p* < 0.001, *ɳ*^2^_p_ = 0.577. The number of recited words in children with DS and TD-MA children did not differ under ST or DT condition (*p* = 0.212), again with TD-CA children naming the largest number of words.

**Figure 2:**
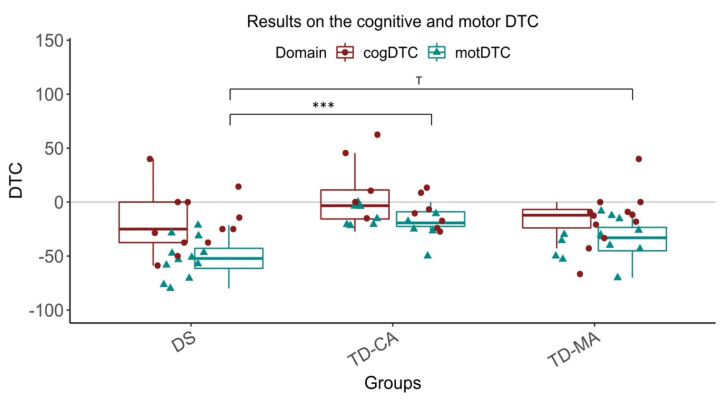
Results on the cognitive and motor DTC in DS, TD-MA, and TD-CA children (*** *p* < 0.001; *T* < 0.10). Note: cogDTC = cognitive dual-task costs; motDTC= motor dual-task costs; DS = Down Syndrome; TD-MA = Typically developing children of the same mental age; TD-CA = typically developing children of the same chronological age.

Regarding the DTC, no significant group differences were observed in cognitive DTC, *F*(2,33) = 2.27, *p* = 0.119, *ɳ*^2^_p_ = 0.121, but group differences were observed in motor DTC, *F*(2,33) = 12.1, *p* < 0.001, *ɳ*^2^_p_ = 0.424. Children with DS differed significantly from TD-CA children (*p* < 0.001) and tended to differ significantly from TD-MA children (*p* = 0.05). Thus, significantly higher cognitive DTC were observed for children with DS. TD-MA and TD-CA children only tended to differ from each other without statistical significance (*p* = 0.065) (Figure 2).

## 4. Discussion

The study investigated motor-cognitive interferences in children with DS and compared them with TD children-matched by mental or chronological age. All groups showed lower performances under the DT condition than the single-task (ST) condition. Children with DS appeared to have difficulties in all conditions, both in motor and cognitive ST and DT conditions. Concerning the DTC, difficulties are mainly observed with the motor task, with motor DTC being greater than cognitive DTC.

In particular, children with motor and/or cognitive impairments exhibited increased difficulties in the parallel processing of motor and cognitive tasks compared to TD children due to the reduced resources in the respective domain (e.g., [18,19] in children with DCD). Our behavioral data are thus in line with the “Cross-Domain Competition Model” [20], showing that two-in their structure-seemingly independent tasks interfere with each other.

To date, there are only two studies on individuals with DS examining motor-cognitive interference in adolescents [12] or young adults [13]. Due to the lack of studies, it is difficult to draw definitive conclusions about motor-cognitive interference in children with DS. However, our findings show that children with DS have increased difficulties in both motor (walking distance covered) and cognitive (number of words recited) performance. In general, the main characteristics of individuals with DS are deficits in verbal skills [10,21] (crucial for the processing of verbal fluency tasks), impairments in EF [22], and cognitive developmental delays ranging from mild to severe intellectual disability [23].

It also appears that children in all groups direct their attentional focus towards the cognitive task and neglect the motor task (Posture Second Strategy). Especially for children with DS, a Posture First Strategy would have been expected to complete the task due to the known difficulties in walking/balance [24]. Some studies reported even an improvement in cognitive performance under DT conditions in TD-CA children [25]. As expected, children with DS showed the highest cognitive and motor DTCs, followed by TD-MA and TD-CA children with the lowest DTCs. These results are consistent with Hagmann-von Arx et al.’s [26] study, demonstrating that motor DTC decreases with increasing age. Accordingly, EF plays a decisive role in walking, especially in children with intellectual disabilities, like DS. The neural network responsible for EF is no longer available for motor performance under DT conditions by allocating resources to the cognitive task. EFs are primarily characterized by high rates of change in the transition period between childhood and adolescence, which may further explain the problems under DT in the sample studied in our study. In general, developmental changes in the functional neuronal connectivity of the brain in children are associated with two general principles that complicate the performance of demanding motor and cognitive tasks or DTs. First, the interaction of neuronal structures in children changes from a predominantly local interaction to an extended interaction of neuronal structures as development proceeds. Second, this development-dependent change in functional connectivity occurs through the segregation of local connections and integration of these areas into previously disparate subnetworks [27]. It would be interesting to see whether these developmental principles also apply to children with DS or whether these principles differ from those TD children.

However, the exact cognitive mechanisms critical for locomotion are not known in detail, especially since some studies could not report reduced performance under DT conditions. The interpretation of the study results is considerably more difficult due to the different methodological procedures, the heterogeneous study protocols, and the partly inconsistent data situation. Also, DTCs are not reported in all studies. Moreover, because the different components of EF show different developmental trajectories [28], the results strongly depend on the choice of cognitive and motor tasks.

Finally, some limitations and strengths of the study should be mentioned. Although the sample size is similar to previous studies (e.g., [13,22]), the overall generalizability is limited by the number of participants. On the one hand, the cross-sectional design also warrants that the results have to be interpreted with caution. On the other hand, the cross-sectional design of this study could be evaluated as an adequate approach providing data in this little investigated field. Future studies need to examine the validity and reliability of comparable dual-task designs and their psychometric properties (intraclass-correlation coefficients, ICC; standard error of measurement, SEM; minimal detectable change, MDC) as it is critical to ensure that the measurement error is smaller than the observed dual-task effect (see a related study in young and old adults [29]). Furthermore, the concurrent inclusion of TD-MA and TD-CA children could be mentioned as a strength.

## 5. Conclusions

With our study results, we confirmed the previously existing findings on DTCs in individuals with DS. As expected, children with DS show the highest cognitive and motor DTCs compared to the other groups. One particularly interesting finding is that all groups seemed to have “chosen” the Posture Second Strategy, in which the attentional focus was on the cognitive task. According to Higuchi [30], the degree of automatization of motor locomotion tasks also depends strongly on the difficulty of the walking task. Young children rarely walk in a straight line. Many paths are winding and curved in everyday tasks, and walking is often interrupted by frequent starts and stops, including many gait patterns with 1 to 3 small steps. Walking with direction changes also requires a necessary asymmetrical placement of the feet and controlling the body in different directions. Thus, walking with a change of direction seems to be an ecologically valid alternative with an increased level of difficulty, which should be used in future studies with children [11].

## Figures and Tables

**Table 1:** Age and sex distribution of children with DS, TD-CA, and TD-MA controls, including mean values (standard deviation).

	DS	TD-CA	TD-MA	Statistical Analyses
	(*n* = 12)	(*n* = 12)	(*n* = 12)	
Age (years)	10.5 ± 1.08 ^§^	10.5 ± 1.07	5.98 ± 1.21 ^#^	*F*(2,33) = 65.8, *p* < 0.001, *η*^2^_p_ = 0.799
Sex (% female)	50.0	50.0	50.0	*CHI*^2^(2) = 0.00, *p* = 1.00

Note. DS = Down Syndrome; TD-CA = Typically developed chronological age; TD-MA = Typically developed - mental age; § Significant difference to MA-adjusted group (*p* < 0.05); # Significant difference to CA-adjusted group (*p* < 0.05; A TD child was assigned to the TD-CA group if the CA was within the 4-month range of the children with DS. A TD child was assigned to the TD-MA control group if the raw score in the PPVT-IV was less than 4 standard deviation points away from the corresponding mean score of the children with DS.

## Data Availability

All relevant data are within the study, and raw data are available on request.
